# Astragaloside IV exhibits anti-tumor function in gastric cancer via targeting circRNA dihydrolipoamide S-succinyltransferase (circDLST)/miR-489-3p/ eukaryotic translation initiation factor 4A1(EIF4A1) pathway

**DOI:** 10.1080/21655979.2022.2063664

**Published:** 2022-04-17

**Authors:** Fagen Li, Ke Cao, Maoyun Wang, Yi Liu, Yin Zhang

**Affiliations:** Senior Department of Traditional Chinese Medicine, the Sixth Medical Center of PLA General Hospital, Beijing, China

**Keywords:** Astragaloside IV, gastric cancer, circDLST, miR-489-3p, EIF4A1

## Abstract

Astragaloside IV (AS-IV) is an inartificial saponin separated from astragalus membranaceus, which has exhibited key anti-tumor regulation in some cancers. Circular RNAs (circRNAs) are important regulators in malignant development of gastric cancer (GC). Herein, we focused on the molecular mechanism of AS-IV with circRNA dihydrolipoamide S-succinyltransferase (circDLST) in GC. CircDLST, microRNA-489-3p (miR-489-3p), and eukaryotic translation initiation factor 4A1 (EIF4A1) levels were detected by quantitative real-time polymerase-chain reaction and western blot. Cell functions were assessed by cell counting kit-8 assay, ethynyl-2’-deoxyuridine assay, colony formation assay, and transwell assay. The interaction between miR-489-3p and circDLST or EIF4A1 was analyzed by dual-luciferase reporter assay. Xenograft tumor assay was adopted to check the role of circDLST and AS-IV *in vivo*. CircDLST and EIF4A1 were upregulated but miR-489-3p was downregulated in GC cells. AS-IV restrained cell proliferation and metastasis in GC cells by downregulating circDLST. CircDLST served as a miR-489-3p sponge, and miR-489-3p inhibition reversed anti-tumor function of AS-IV. EIF4A1 was a target for miR-489-3p and circDLST sponged miR-489-3p to regulate EIF4A1. AS-IV suppressed GC cell progression via circDLST-mediated downregulation of EIF4A1. Also, AS-IV recued tumor growth *in vivo* via targeting circDLST to regulate miR-489-3p/EIF4A1 axis. AS-IV inhibited the development of GC through circDLST/miR-489-3p/EIF4A1 axis.

## GRAPHICAL ABSTRACT

**Figure:**
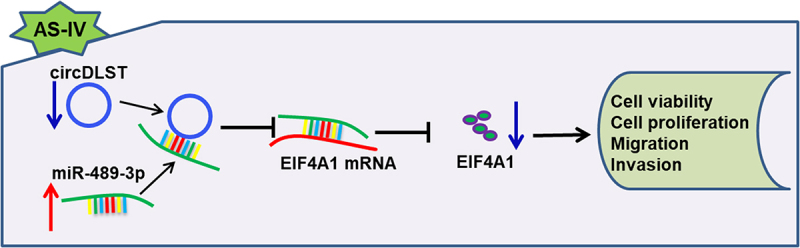

## Highlights

1. AS-IV restrained the development of GC cells.
2. The abundance of circDLST was upregulated in GC cells.
3. CircDLST stimulated the progression of GC cells.
4. CircDLST expedited the advancement of GC via miR-489-3p.
5. MiR-489-3p repressed GC progression by targeting EIF4A1.

## 1 Introduction

Gastric cancer (GC) is a prevalent cancer in East Asia [1], and the overall five-year survival rate was only about 20% [2]. There are many factors affecting GC, such as age, sex, smoking, and *Helicobacter pylori* infection [3]. Surgery and chemotherapy are still the main treatment methods, but the prognosis is not satisfactory. Astragaloside IV (AS-IV) is a traditional Chinese medicine with important protection for digestive and nervous systems [4]. AS-IV has exerted anti-cancer regulation in various cell behaviors of GC, including proliferation, migration, and angiogenesis [5]. But the functional mechanism of AS-IV in GC is not fully addressed.

Circular RNAs (circRNAs) acted as key factors in different diseases [6,7]. CircRNA_100269 suppressed tumor cell growth of GC [8] and circ_100782 promoted cell metastasis in GC [9]. CircRNA cullin 2 (circCUL2) regulated cisplatin sensitivity in GC cells [10]. In addition, circRNA dihydrolipoamide S-succinyltransferase (circDLST) stimulated tumor growth in GC [11]. However, the association of AS-IV with circRNA is not clear and this study focused on AS-IV function with circDLST in GC.

CircRNAs can serve as miRNA sponges to regulate gene levels in affecting cancer biology [12]. The oncogenic function of circDLST has been indicated to be achieved by targeting miR-502-5p/NRAS axis [11]. MiR-489-3p has tumor-inhibitory role in a variety of tumors, such as melanoma [13], bladder cancer [14], and glioblastoma [15]. MicroRNAs (miRNAs) take part in affecting genes to modulate cell processes [16,17]. Also, miR-489-3p inhibited cell proliferation and migration in GC by targeting HDCA7 and PROX1 [18,19]. The eukaryotic translation initiation factor 4A1 (EIF4A1) was an important regulator of many cancers [20]. For instance, inhibition of EIF4A1 enhanced cell apoptosis and reduced tumor growth in breast cancer and prostate cancer [21,22]. Li *et al*. reported that EIF4A1 functioned as an oncogene in GC, and circ_0008035 promoted GC progression by upregulating EIF4A1 via sponging miR-599 [23]. Our prediction showed that there were binding sites between circDLST and miR-489-3p, as well as EIF4A1 and miR-489-3p. Thus, the potential of circDLST in regulating EIF4A1 by binding to miR-489-3p is worth studying in GC.

In this work, we hypothesized that circDLST could target miR-489-3p to regulate EIF4A1 expression. The goals of this study were to explore the functions of circDLST in regulating AS-IV-induced tumor inhibition in GC, as well as its molecular mechanism with miR-489-3p and EIF4A1.

## 2 Materials and methods

### 2.1. Cell lines and transfection

GC cell lines (HGC-27 and MKN-45) were used for the current research, with GES cells as negative control. All cells were acquired from the JCRB cell bank (Osaka, Japan) and cultured with 5% CO_2_. HGC-27 and MKN-45 cells were exposed to AS-IV (0, 10, 20, 40 μg/mL, Sigma-Aldrich, St Louis, MO, USA). The circDLST vector (circDLST), the control (vector); miR-489-3p mimic (miR-489-3p, miR10002805-1-5), mimic control (miR-NC, miR1N0000001-1-5), miR-489-3p inhibitor (in-miR-489-3p, miR20002805-1-5), inhibitor control (in-miR-NC, miR2N0000001-1-5); Bio-miR-NC, Bio-miR-489-3p; small interfering RNA (siRNA) of EIF4A1 (si-EIF4A1) and siRNA control (si-NC) were offered by Ribobio (Guangzhou, China). Transfection was carried out by Lipofectamine 2000 (11,668,019; Invitrogen, Carlsbad, CA, USA).

### 2.2. Quantitative real-time polymerase chain reaction (qRT-PCR)

RNAs were extracted from GC cells via Trizol (Takara, Shiga, Japan); then, 1 µg RNA of each sample was applied to prepare cDNA. Subsequently, TB Green kit (RR430A; Takara) was adopted to implement qRT-PCR. Glyceraldehyde-phosphate dehydrogenase (GAPDH) and RNU6 (U6) were used as housekeeping genes. The primers were listed in Table 1. The relative expression levels were computed by the 2^−ΔΔCt^ method [24].

**Table 1.** Primer sequences used for qRT-PCR

Name		Primers (5’-3’)
circDLST	Forward	CAAAACCCCAGCGTTTGCAG
	Reverse	CACTGTTGTTAATGCTTTCTCCCA
miR-489-3p	Forward	GTATGAGTGACATCACATATAG
	Reverse	CAGTGCGTGTCGTGGAGT
EIF4A1	Forward	TCATCAACACCCGGAGGAAG
	Reverse	TGCACATCAATGCCTCTGGC
GAPDH	Forward	TCCCATCACCATCTTCCAGG
	Reverse	GATGACCCTTTTGGCTCCC
U6	Forward	CTCGCTTCGGCAGCACATATACT
	Reverse	ACGCTTCACGAATTTGCGTGTC

### 2.3. Western blot

The protein detection was performed by western blot as previously described [25]. GC cells were exposed to a RIPA buffer (R0278; Sigma) for total protein extraction. Then, protein products were prepared and separated through a 10% sodium dodecyl sulfate polyacrylamide gel electrophoresis (SDS-PAGE), followed by protein transferring to polyvinylidene fluoride membranes (Sigma). The membranes were incubated with the primary antibodies: anti-EIF4A1 (ab185946; 1:10,000; Abcam, Cambridge, MA, USA), anti-E-cadherin (ab40772; 1:10,000; Abcam), anti-N-cadherin (ab76011; 1:1,000; Abcam), anti-Vimentin (ab92547; 1:1,000; Abcam), and anti-β-actin (ab8227; 1:1000; Abcam). Then, a secondary antibody (ab205718; 1:2500; Abcam) was incubated at room temperature for 1 h, and the bands were photographed.

### 2.4. CCK8 assay

GC cells were seeded in 96-well plates overnight and treated with AS-IV or transfected with different substances. Then, cells were incubated with CCK8 (20 µL/well, 96,992; Sigma). After 4 h, the absorbance at 450 nm was examined via a microplate reader (Thermo Fisher Scientific, Waltham, MA, USA).

### 2.5. Cell proliferation assay

Cell proliferation ability was measured by EdU assay [26]. The EdU Apollo Imaging Kit (RiboBio) was used in this study. Fifty μM/well EdU was added, and then GC cells were exposed to 4% paraformaldehyde for 0.5 h. Next, cells were incubated with Apollo solution (RiboBio) and diamidine phenylindole (DAPI; RiboBio). Cell pictures were saved by a microscopy (20 × objective lenses).

### 2.6. Colony formation assay

GC (1 × 10^6^) cells were cultured in 6-well plates for 2 weeks. Whereafter, the white cell colonies were fixed through methanol (Sigma) and stained through crystal violet (0.5%, Sigma). The colonies were numbered in a manual way.

### 2.7. Transwell assay

Cell motility was analyzed using transwell assay [27]. Herein, GC cells were determined by a transwell chamber with 8 μm pore polycarbonate membrane (BD Bio-sciences, Bedford, MA, USA). In brief, 4 × 10^5^ transfected GC cells in 100 µL serum-free medium were planted into the top chamber. Then, the lower chamber was added with 500 µL of DMEM and 10% FBS. The migrated cells on the reverse side of the membrane were stained. To measure the cell invasion, the transwell chamber must be first coated with matrigel (BD Biosciences). Eventually, migrated and invaded cells were counted under a microscope (Thermo Fisher Scientific).

### 2.8. Dual-luciferase reporter assay

The conjunct sites between miR-489-3p and circDLST or EIF4A1 were predicted by starbase (http://starbase.sysu.edu.cn/). Then, circDLST or EIF4A1 wild type and mutant plasmids (circDLST-WT, EIF4A1-3’UTR-WT or circDLST-MUT, EIF4A1-3’UTR-MUT) were offered by Ribobio. Each plasmid was co-transfected with miR-NC or miR-489-3p for 48 h, and then the luciferase activity was evaluated using dual-luciferase reporter kit (E2920; Promega, Madison, WI, USA).

### 2.9. RNA immunoprecipitation (RIP) assay

RIP assay was adopted through an Imprint® RNA RIP kit (RIP-12RXN; Sigma) to analyze the interaction between miR-489-3p and circDLST. GC cells were incubated with magnetic beads of anti-Argonaute2 or anti-IgG group for 12 h. After the RNA was isolated, miR-489-3p and circDLST levels were assayed by qRT-PCR.

### 2.10. RNA pull-down assay

GC cells were lysed and incubated with the probe-bead compound for 3 h. Next, the protein and DNA were removed from beads. RNA was separated by a RNeasy Mini Kit (Sigma), and qRT-PCR was used for quantification of miR-489-3p and circDLST.

### 2.11. Xenograft models

The animal assay was performed with the supervision of the Animal Care and Use Committee of the Chinese PLA General Hospital. Eighteen nude mice (6 weeks; 18–22 g; female) were purchased from Beijing Vital River Laboratory Animal Technology Co., Ltd. (Beijing, China). These mice were separated into three groups: control+vector, AS-IV+vector, AS-IV+circDLST. Mice were hypodermically vaccinated with 4 × 10^6^ HGC-27 cells with transfection vector or circDLST and then injected with 0.3 mL AS-IV (40 mg/kg) for 21 consecutive days. Tumor volume = length × width^2^ × 0.5. After 28 days, the animals were euthanized, and the tumor tissues were weighed. Total RNA and protein were extracted for expression detection.

### 2.12. Immunohistochemistry (IHC) assay

Ki67 protein expression was determined using the IHC assay [28]. Firstly, the tissues were utilized to prepare paraffin slides. The slides were repaired by antigen and nurtured with anti-Ki67 (ab92742; 1:1,000; Abcam). Afterward, the sections were hatched with secondary antibody (ab205718; 1:1,000; Abcam), and the sections were photographed.

### 2.13. Statistical assay

All statistical data were collected from three repetitions. The difference was assessed by the Student’s *t*-test or analysis of variance (ANOVA) through GraphPad Prism 8. *P* < 0.05 was defined as a significant difference.

## 3 Results

### 3.1. AS-IV restrained cell proliferation and metastasis in GC cells

**Figure 1.**
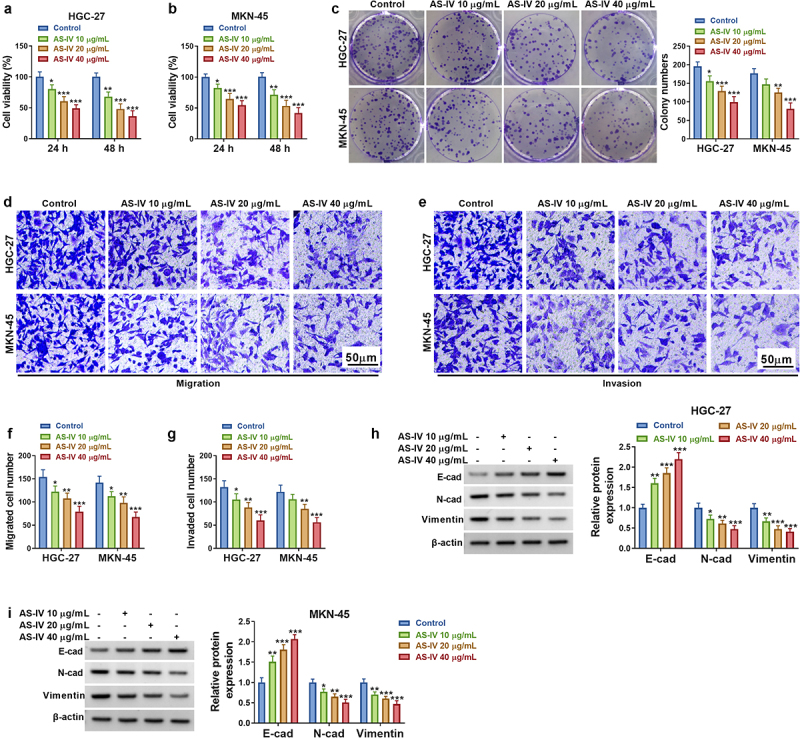
AS-IV restrained cell proliferation and metastasis in GC cells. (a and b) CCK8 assay was applied to evaluate the cell viability cells. (c) The colony formation assay unfolded the number of colonies. (d-g) The transwell assay assessed the cell migration and invaded. (h and i) The protein levels of E-cad, N-cad, and Vimentin were examined by western blot. *P < 0.05, **P < 0.01, ***P < 0.001.

CircRNAs are usually dysregulated in cancer and regulated GC progression via miRNA/mRNA axis. AS-IV has been identified to act as a tumor inhibitor in GC by targeting molecular pathways. The goals of this study were to explore the association of circDLST with AS-IV in GC. In addition, circDLST was hypothesized to target miR-489-3p to regulate EIF4A1 expression. Firstly, the anti-tumor function of AS-IV was affirmed in our cell lines. CCK-8 assay showed that AS-IV decreased cell vitality in a dose-dependent way (Figure 1(a,b)). Colony formation assay unfolded that AS-IV significantly lessened the number of colonies of GC cells (Figure 1(c)). Besides, transwell assay illustrated that AS-IV suppressed invasion and migration abilities in HGC-27 and MKN-45 cells (Figure 1(d-g)). E-cadherin, N-cadherin, and Vimentin are important markers in regulating cell migration and invasion. AS-IV conspicuously downregulated the protein levels of N-cadherin and Vimentin but increased E-cadherin protein expression (Figure 1(h,i)). Our results indicated that AS-IV restrained cell proliferation and metastasis in GC cells.

### 3.2. CircDLST overexpression restored AS-IV induced inhibition in GC cells

**Figure 2.**
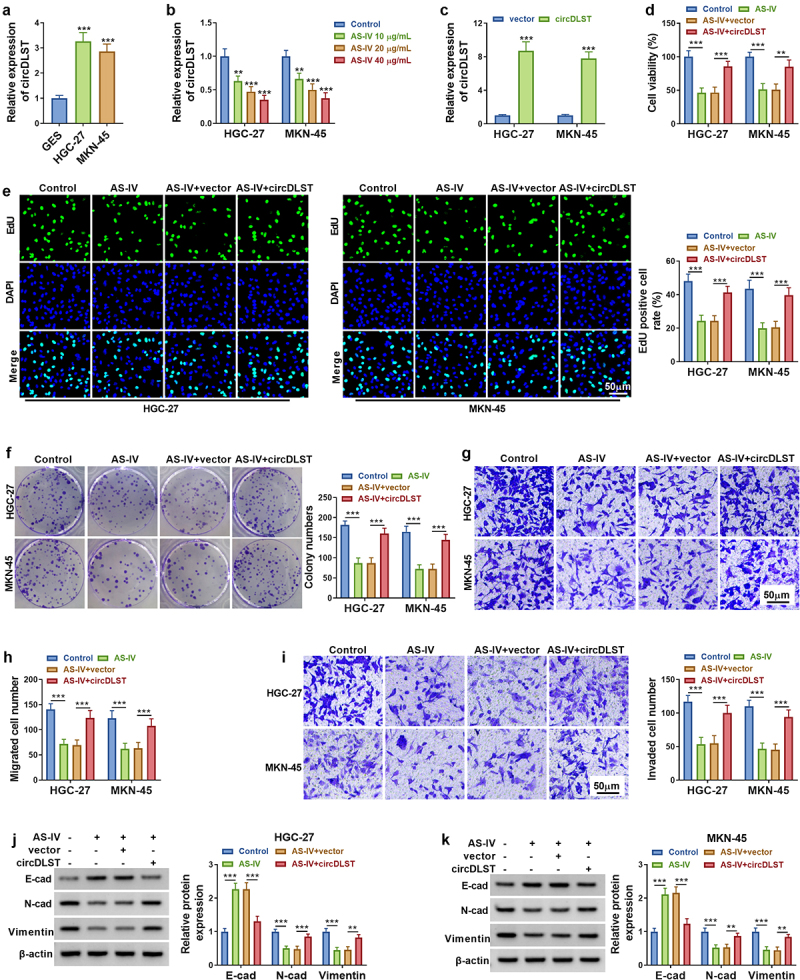
CircDLST overexpression restored AS-IV induced inhibition in GC cells. (a). The expression of circDLST was distinguished by qRT-PCR. (b) The relative levels of circDLST were measured by qRT-PCR. (c) The level circDLST was evaluated by qRT-PCR. (d-f) The cell proliferation. (g and h) The cell migration. (i) The cell invaded. (j and k) The protein levels of E-cad, N-cad, and Vimentin were evaluated. **P < 0.01, ***P < 0.001.

The circDLST expression was much higher in HGC-27 and MKN-45 cells (Figure 2(a)). In addition, AS-IV induced significant downregulation of circDLST in GC cells (Figure 2(b)). Then, circDLST was evidently upregulated by circDLST transfection in GC cells versus the vector group (Figure 2(c)). Functionally, AS-IV-induced cell viability inhibition was attenuated after transfection with circDLST (Figure 2(d)). The inhibitory functions of AS-IV in cell proliferation (Figure 2(e,f)) and migration/invasion (Figure 2(g-i)) were also reversed by overexpression of circDLST. In addition, circDLST mitigated the protein-level changes of E-cadherin, N-cadherin, and Vimentin by AS-IV in HGC-27 and MKN-45 cells (Figure 2(j,k)). Our consequences demonstrated that circDLST relieved anti-tumor function of AS-IV in GC cells.

### 3.3 MiR-489-3p targeted circDLST

**Figure 3.**
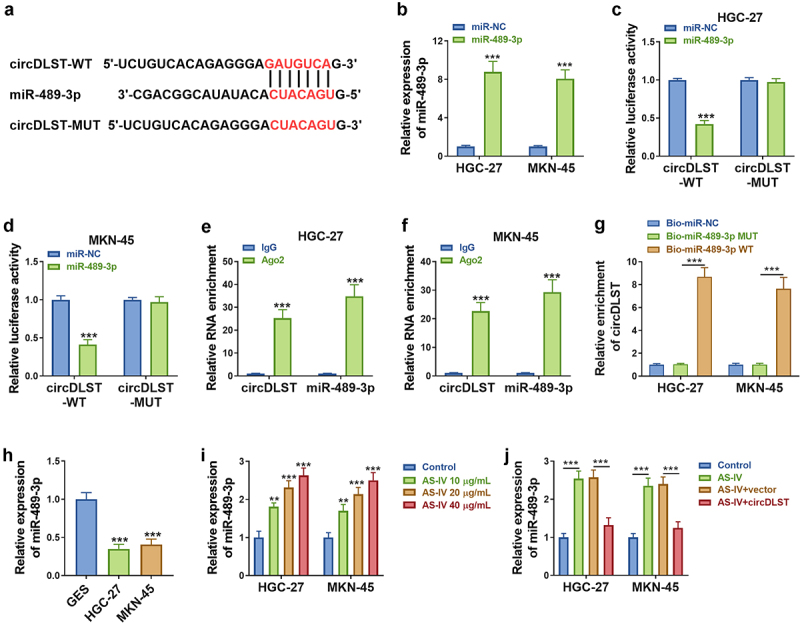
CircDLST served as a sponge for miR-489-3p. (a). The conjoint miRNAs of circDLST were predicted. (B) The miR-489-3p content in GC cells was detected by qRT-PCR. (c and d) The relationship between circDLST and miR-489-3p was assessed. (e and f) RIP assay was utilized to authenticate the link between circDLST and miR-489-3p. (g) RNA pull-down assay was adopted to confirm the link between circDLST and miR-489-3p. (h-j) The content of miR-489-3p in GC cells was uncovered by qRT-PCR. **P < 0.01, ***P < 0.001.

Starbase predicted that miR-489-3p might be a target of circDLST (Figure 3(a)). Besides, miR-489-3p was markedly overexpressed by miR-489-3p transfection in GC cells compared to miR-NC transfection (Figure 3(b)). The luciferase activity was reduced in circDLST-WT and miR-489-3p (relative to miR-NC) co-transfected GC cells, but there was no alteration of luciferase activity in circDLST-MUT plasmid (Figure 3(c,d)). The RIP assay and RNA pull-down assay further suggested the interaction between miR-489-3p and circDLST in GC cells (Figure 3(e-g)). Furthermore, qRT-PCR showed the downregulation of miR-489-3p in GC cells compared with normal GSE cells (Figure 3(h)). Moreover, AS-IV treatment resulted in upregulation of miR-489-3p (Figure 3(i)) and this effect was alleviated by circDLST (Figure 3(j)). Thus, circDLST targeted miR-489-3p in GC.

### 3.4 MiR-489-3p downregulation reversed AS-IV-induced inhibition in GC cells

**Figure 4.**
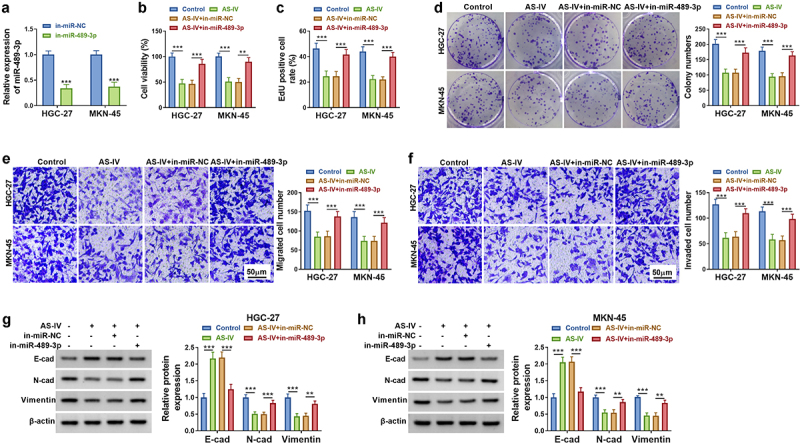
MiR-489-3p downregulation reversed AS-IV-induced inhibition in GC cells. (a). The miR-489-3p content was distinguished by qRT-PCR. (b-d) The cell proliferation. (e) The cell migration. (f) The cell invaded. (g and h) The protein levels of E-cad, N-cad, and Vimentin were quantified. **P < 0.01, ***P < 0.001.

The inhibiting efficiency of in-miR-489-3p transfection for miR-489-3p level was conspicuous in GC cells (Figure 4(a)). Cell experiments manifested that miR-489-3p inhibitor reversed the suppressive regulation of AS-IV in cell viability (Figure 4(b)), proliferation (Figure 4(c,d)), migration (Figure 4(e)) and invasion (Figure 4(f)) in HGC-27 and MKN-45 cells. Western blot data showed that E-cadherin protein level was reduced but N-cadherin and Vimentin levels were increased in AS-IV+in-miR-489-3p group relative to AS-IV+in-miR-NC group (Figure 4(g,h)). These findings suggested that AS-IV inhibited GC cell progression partly by upregulating miR-489-3p.

### 3.5 CircDLST targeted miR-489-3p to induce expression change of EIF4A1 in GC cells

**Figure 5.**
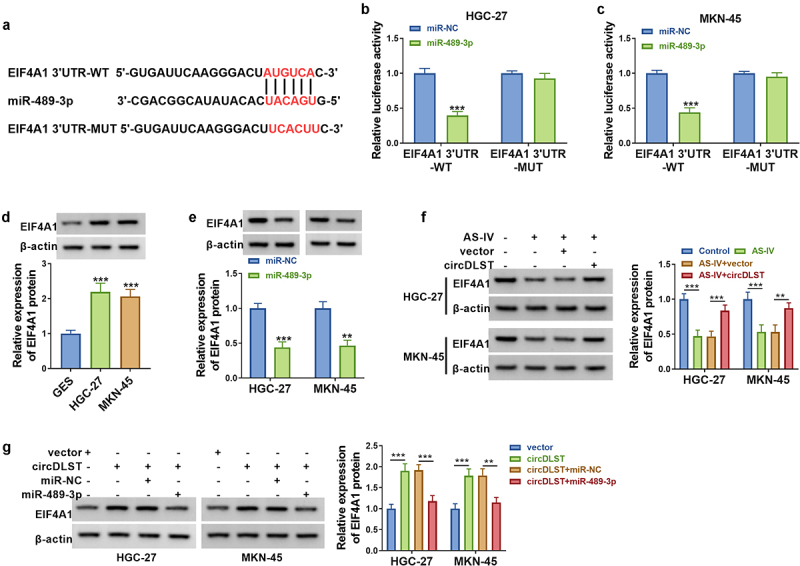
CircDLST targeted miR-489-3p to induce expression change of EIF4A1 in GC cells. (a) The conjunct site between miR-489-3p and EIF4A1 was scrutinized by starbase. (b and c) The link between miR-489-3p and EIF4A1 was confirmed. (d-g) The content of EIF4A1 was distinguished by western blot. **P < 0.01, ***P < 0.001.

Starbase was adopted to predict the conjoint sites of miR-489-3p in EIF4A1 3ʹUzR (Figure 5(a)). The luciferase activity of EIF4A1 3ʹUTR-WT was reduced by miR-489-3p overexpression, but there was no difference in the EIF4A1 3ʹUTR-MUT group (Figure 5(b,c)). EIF4A1 protein levels were upregulated in HGC-27 and MKN-45 cells contrasted to GSE cells (Figure 5(d)). Besides, the protein expression of EIF4A1 was reduced by miR-489-3p upregulation in GC cells (Figure 5(e)). Additionally, AS-IV-induced EIF4A1 protein downregulation was attenuated after transfection with circDLST (Figure 5(f)). Moreover, circDLST upregulated protein level of EIF4A1 and this change was counteracted by miR-489-3p transfection (Figure 5(g)). Altogether, circDLST could regulate EIF4A1 via interacting with miR-489-3p.

### 3.6. CircDLST reversed anti-tumor function of AS-IV in GC cells via upregulating EIF4A1

**Figure 6.**
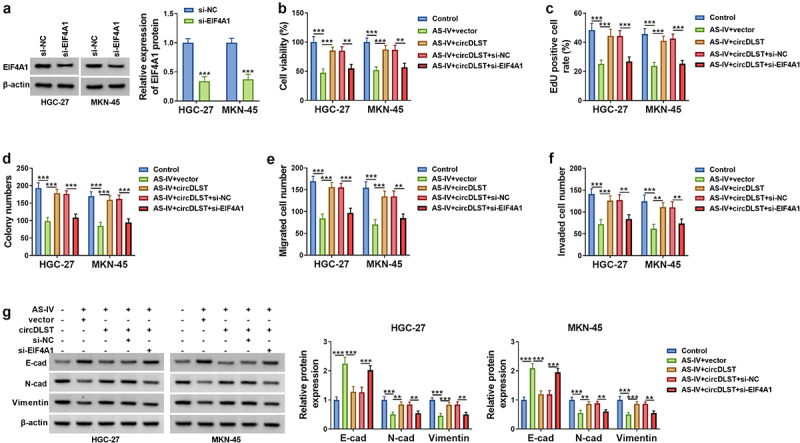
CircDLST reversed anti-tumor function of AS-IV in GC cells via upregulating EIF4A1. (a) The abundance of EIF4A1 was assessed by western blot. (b-d) The cell proliferation. (e) The cell migration. (f) The cell invaded. (g and h) The contents of E-cad, N-cad, and Vimentin were scrutinized. **P < 0.01, ***P < 0.001.

EIF4A1 expression was markedly decreased by si-EIF4A1 in GC cells, relative to si-NC group (Figure 6(a)). All attenuated effects of circDLST on cell viability (Figure 6(b)), proliferation (Figure 6(c,d)), migration (Figure 6(e)) and invasion (Figure 6(f)) in AS-IV-treated cells were partly abolished after knockdown of EIF4A1. The circDLST-induced restoration of E-cadherin, N-cadherin, and Vimentin protein expressions was also offset by si-EIF4A1 in GC cells with AS-IV treatment (Figure 6(g)). In brief, circDLST regulated the function of AS-IV via upregulating EIF4A1.

### 3.7. AS-IV inhibited tumor growth by regulating circDLST/miR-489-3p/EIF4A1

**Figure 7.**
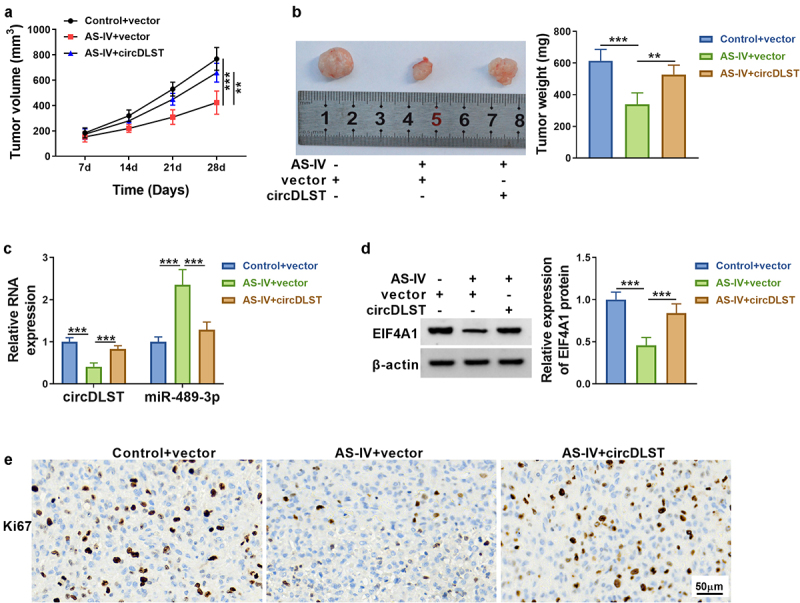
AS-IV inhibited tumor growth by regulating circDLST/miR-489-3p/EIF4A1. (a). Tumor growth was detected. (b) Tumor weight was measured. (c and d) The circDLST, miR-489-3p, and EIF4A1 contents were revealed by qRT-PCR and western blot. (e) IHC was enforced to scrutinize the Ki-67 abundance. **P < 0.01, ***P < 0.001.

As revealed in Figure 7(a,b), AS-IV blocked tumor volume and weight, but circDLST overexpression could lessen tumor growth inhibition. Additionally, circDLST and EIF4A1 were upregulated but miR-489-3p was notably increased in AS-IV+circDLST group compared with AS-IV+vector group (Figure 7(c,d)). The consequences from IHC indicated that AS-IV treatment reduced Ki67 protein level and circDLST upregulation abrogated this effect in tumor tissues (Figure 7(e)). These results affirmed that AS-IV reduced tumor growth via targeting circDLST/miR-489-3p/EIF4A1 axis.

## 4 Discussion

Herein, AS-IV was shown to impede GC progression by targeting circDLST to mediate miR-489-3p/EIF4A1 axis. This study provided a clear functional mechanism underlying AS-IV in GC.

Some studies have discovered that AS-IV could regulate the progression of diseases. For example, AS-IV inhibited lung cancer metastasis [29] and AS-IV took part in podocyte apoptosis of diabetic nephropathy [30]. Also, the previous reports indicated that AS-IV attenuated hypoxia/reoxygenation injury-induced cell apoptosis and inhibited cell progression of lung cancer [31,32]. However, there was no research for AS-IV in GC. Our consequences demonstrated that AS-IV restrained cell proliferation and metastasis in GC cells. Thus, AS-IV also exhibited anti-tumor function in GC. AS-IV can affect the progression of diseases by acting on RNAs. In this study, circDLST overexpression significantly relieved the inhibitory regulation of AS-IV in GC cells. These findings suggested that AS-IV inhibited GC development by downregulating circDLST.

The previous study has shown the oncogenic role of circDLST in GC [33]. CircDLST attenuated the inhibition of AS-IV in malignant behaviors, confirming that circDLST promoted the progression of GC cells. Noncoding RNAs have exhibited target interaction with miRNAs, thus regulating disease progression [34,35]. CircDLST has been reported to act as a sponge of miR-502-5p in gastric cancer [33]. Herein, online prediction and target analysis showed that miR-489-3p was a miRNA target for circDLST.

MiR-489-3p impeded the progression of melanoma, bladder cancer, and renal cell carcinoma [13,14,36]. Our data manifested that miR-489-3p was downregulated in GC, which was consistent with the results of Zhang *et al*. [19]. Moreover, rescue experiments indicated that AS-IV-induced tumor inhibition in GC was related to upregulation of miR-489-3p. More importantly, EIF4A1 was identified as a downstream gene of miR-489-3p and circDLST upregulated EIF4A1 via sponging miR-489-3p in GC cells.

EIF4A1 played a key role in different cancers, such as lymphoma, breast cancer, and prostate cancer [21,22]. Gao *et al*. reported that EIF4A1 accelerated cell migration and invasion in GC [37]. Currently, EIF4A1 knockdown reversed the oncogenic function of circDLST in AS-IV-treated GC cells. These results affirmed that EIF4A1 served as an oncogene in GC and AS-IV inhibited GC progression via downregulating circDLST to reduce EIF4A1 level. Therefore, AS-IV function was achieved partly by regulating circDLST/miR-489-3p/EIF4A1 axis in GC cells. *In vivo* assay further suggested that AS-IV suppressed tumor growth via targeting circDLST to regulate miR-489-3p and EIF4A1 levels.

Given the high expression of circDLST in GC cells, circDLST might be used as a diagnostic target for GC. In addition, circDLST attenuated the tumor inhibition by AS-IV in GC progression. Thus, circDLST had the potential to act as a biomarker in the treatment process of AS-IV for GC.

## 5 Conclusion

In conclusion, our results first uncovered that AS-IV inhibited the progression of GC via targeting circDLST/miR-489-3p/EIF4A1 axis (Graphical abstract). This study might contribute to understanding the anti-tumor mechanism of AS-IV in GC.
